# Development of a Digital Health Intervention to Support Patients on a Waitlist for Orthopedic Specialist Care: Co-Design Study

**DOI:** 10.2196/41974

**Published:** 2023-12-08

**Authors:** Alexander Tacey, Jack Behne, Rhiannon K Patten, Minh Truc Ngo, Rees Thomas, Jessica Ancilleri, Chelsea Bone, Angela Paredes Castro, Helen McCarthy, Katherine Harkin, Julia FM Gilmartin-Thomas, Amir Takla, Calum Downie, Jane Mulcahy, Michelle Ball, Jenny Sharples, Sarah Dash, Amy Lawton, Breanna Wright, Peter Sleeth, Tina Kostecki, Christopher Sonn, Michael J McKenna, Vasso Apostolopoulos, Rebecca Lane, Catherine M Said, Mary De Gori, Andrew McAinch, Phong Tran, Itamar Levinger, Alexandra Parker, Mary N Woessner, Michaela Pascoe

**Affiliations:** 1 Institute for Health and Sport (iHeS) Victoria University Melbourne Australia; 2 Department of Orthopaedic Surgey Western Health Melbourne Australia; 3 Department of Physiotherapy Western Health Melbourne Australia; 4 College of Health and Biomedicine Victoria University Melbourne Australia; 5 First Year College Victoria University Melbourne Australia; 6 School of Public Health and Preventive Medicine Monash University Melbourne, Vic Australia; 7 Australian Institute for Musculoskeletal Science (AIMSS) Melbourne Australia; 8 Department of Medicine, Western Health The University of Melbourne Melbourne Australia; 9 Australian Sports Physiotherapy Melbourne Australia; 10 Department of Health Professions Swinburne University of Technology Melbourne Australia; 11 School of Physiotherapy Melbourne School of Health science The University of Melbourne Melbourne Australia; 12 The Institute for Mental and Physical Health and Clinical Translation (IMPACT) Food & Mood Centre, School of Medicine Barwon Health, Deakin University Geelong Australia; 13 School of Social Sciences University of Tasmania Launceston Australia

**Keywords:** osteoarthritis, web intervention, eHealth, orthopedic waitlist, human-centered design, self-management, knee pain, hip pain, mobile phone

## Abstract

**Background:**

The demand for orthopedic specialist consultations for patients with osteoarthritis in public hospitals is high and continues to grow. Lengthy waiting times are increasingly affecting patients from low socioeconomic and culturally and linguistically diverse backgrounds who are more likely to rely on public health care.

**Objective:**

This study aimed to co-design a digital health intervention for patients with OA who are waiting for an orthopedic specialist consultation at a public health service, which is located in local government areas (LGAs) of identified social and economic disadvantage.

**Methods:**

The stakeholders involved in the co-design process included the research team; end users (patients); clinicians; academic experts; senior hospital staff; and a research, design, and development agency. The iterative co-design process comprised several key phases, including the collation and refinement of evidence-based information by the research team, with assistance from academic experts. Structured interviews with 16 clinicians (female: n=10, 63%; male: n=6, 38%) and 11 end users (age: mean 64.3, SD 7.2 y; female: n=7, 64%; male: n=4, 36%) of 1-hour duration were completed to understand the requirements for the intervention. Weekly workshops were held with key stakeholders throughout development. A different cohort of 15 end users (age: mean 61.5, SD 9.7 y; female: n=12, 80%; male: n=3, 20%) examined the feasibility of the study during a 2-week testing period. The System Usability Scale was used as the primary measure of intervention feasibility.

**Results:**

Overall, 7 content modules were developed and refined over several iterations. Key themes highlighted in the clinician and end user interviews were the diverse characteristics of patients, the hierarchical structure with which patients view health practitioners, the importance of delivering information in multiple formats (written, audio, and visual), and access to patient-centered information as early as possible in the health care journey. All content was translated into Vietnamese, the most widely spoken language following English in the local government areas included in this study. Patients with hip and knee osteoarthritis from culturally and linguistically diverse backgrounds tested the feasibility of the intervention. A mean System Usability Scale score of 82.7 (SD 16) was recorded for the intervention, placing its usability in the excellent category.

**Conclusions:**

Through the co-design process, we developed an evidence-based, holistic, and patient-centered digital health intervention. The intervention was specifically designed to be used by patients from diverse backgrounds, including those with low health, digital, and written literacy levels. The effectiveness of the intervention in improving the physical and mental health of patients will be determined by a high-quality randomized controlled trial.

## Introduction

### Background

Osteoarthritis is a leading cause of disability in older adults globally and has substantial health and economic implications for the individuals affected by it and the health care system [1,2]. It is estimated that 250 million people worldwide are currently affected by osteoarthritis [2]. An aging population and increasing rates of obesity and joint injuries indicate that the prevalence of osteoarthritis will continue to rise and become more burdensome [3]. As a result of the increasing prevalence of osteoarthritis, waitlists for orthopedic specialist appointments are extensive and continuing to grow [4]. The current median waiting time for a consultation in public hospital orthopedic specialist clinics in the state of Victoria, Australia, is 76 days [5]. Although this may not seem excessive, the 90th percentile waiting time is 803 days, which applies to routine patients (category 3), commonly those with osteoarthritis [5]. Many of the patients waiting for >2 years are likely to be from low socioeconomic and culturally and linguistically diverse (CALD) backgrounds who rely on the public health system [6]. By contrast, those who have the means and financial ability to afford private health care often have little to no waiting time for a specialist consultation or surgery.

Physical activity, weight management, and osteoarthritis education are the core conservative (nonsurgical) osteoarthritis management strategies recommended by evidence-based guidelines [7-10]. However, many patients lack access to medical and allied health services because of barriers at the patient, health professional, and health service levels [11]. General practitioners often prescribe pharmacological therapies or refer patients to orthopedic specialists rather than promoting conservative management strategies [12]. In addition, patients’ lived experiences as well as the communication of information by health professionals can promote misconceptions that may be detrimental to disease management [13]. For instance, many patients believe that their osteoarthritis is “bone on bone” and will progressively worsen over time and that physical activity is harmful to their joints [13]. Uncertainty and long waiting times result in many people seeking out digital resources, such as websites, social media, and smartphone apps, to assist them with managing their symptoms [14]. Although the use of the internet to educate patients shows promising potential because of its low cost and broad reach, there is a risk that patients may access information that is both unreliable and irrelevant [15].

The rise in the use of digital resources to access medical information has been accompanied by an increase in the number of digital interventions developed for people with osteoarthritis [16]. These interventions are generally well accepted and result in improvements in pain and physical function [16,17]. However, a notable limitation of the existing interventions is the absence of culturally and linguistically appropriate content and resources designed for people who do not speak the native language of the country in which the interventions were developed [16]. People from CALD populations face many challenges in accessing and receiving appropriate health care, including a lack of translation services, conflicting social and cultural beliefs, and low health literacy levels [6]. Specific to osteoarthritis, it is known that people with low levels of health literacy are generally less likely to engage in the recommended management strategies than those with high levels of health literacy [18]. Thus, to increase the likelihood of a digital self-management intervention being effective, it is important that the intervention be well accepted by end users and specific to the needs of all patients.

Co-design is a process in which the targeted end users and relevant stakeholders form a partnership with researchers to design a product or service together [19]. In Australia, partnering with consumers is embedded into the National Safety and Quality Health Service Standards [20]. In health care, the co-design process has recently gained traction because of its iterative manner, cocreating knowledge from the lived experiences of end users and helping design services around these experiences [21].

The aim of this study was to co-design and test the feasibility of a digital health intervention developed to assist patients with osteoarthritis with the management of their physical and mental health while waiting for an orthopedic specialist consultation at a public health service. In addition, the intervention was designed and developed to suit the population within the local government areas (LGAs) of the western suburbs of Melbourne, Australia. These LGAs have a higher prevalence of people from socially disadvantaged and CALD backgrounds than the rest of Victoria and Australia [22].

### Objectives

The objectives of this co-design study were to (1) identify and engage the stakeholders integral to the co-design of a novel digital health intervention for people with osteoarthritis on an orthopedic waitlist, (2) develop content from the existing evidence-based recommendations and refine the content based on input from academic experts, (3) facilitate structured interviews with end users and clinicians to understand the needs of the population, (4) understand the stakeholders’ expectations of a novel digital health intervention, (5) develop a minimum viable product (MVP) for the digital health intervention incorporating key features and designs, and (6) conduct feasibility testing of the intervention with end users.

## Methods

This study was conducted in the western suburbs of Melbourne, Australia, with patients awaiting specialized care from the orthopedic department at Western Health, a major tertiary health service provider in the region.

### Ethical Considerations

The study was approved by the Melbourne Health Ethics Committee with a mirror approval from the Victoria University Human Research Ethics Committee (MH HREC 2021.055). All the participants provided written informed consent. All study data were deidentified and are reidentifiable only by members of the primary research team who have access to the code. The code and reidentifiable information are stored securely and inaccessible to anybody outside the primary research team. End users who participated in the co-design interviews and feasibility testing were provided with gift cards to the value of Aus $50 (US $32.77) as compensation for their time and involvement. Clinicians involved in the co-design interviews were offered a gift card to the value for Aus $230 (US $150.72) as compensation for their time and involvement.

### Study Design

**Figure 1:**
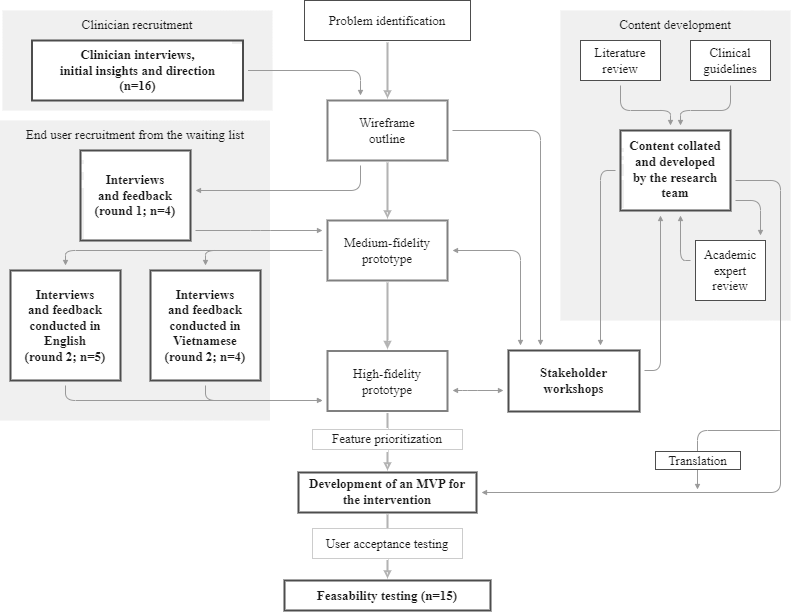
Outline of the co-design process. MVP: minimum viable product.

The co-design process included several key design components, including a wireframe outline, medium-fidelity prototype that provided a basic outline of the intervention with limited functionality, and high-fidelity prototype that provided a realistic outline of the design and user interface of the intervention. The design and development process, involving interviews with end users (patients) and clinicians, stakeholder workshops, and content development, is outlined in Figure 1.

### Problem Identification

Academics and clinicians from the major tertiary education and health service in the western suburbs of Melbourne identified, through collaborative consultation, the need to address the lengthy waiting periods faced by many patients waiting for an orthopedic specialist consultation. The research team identified the need for an evidence-based and patient-centered website that could provide patients with relevant clinical advice about the management of osteoarthritis with low ongoing costs and resource requirements.

### Stakeholder Engagement and Workshops

Initial consultations with key stakeholders began in 2020 and continued throughout the development process until mid-2022. This early engagement informed the focus of and technical requirements for the digital health intervention before commencing the co-design process. In addition to the research team, various stakeholders were included in the consultations, including clinicians and leadership representatives from hospitals and clinical services and academic experts. In addition, a local research, design, and development agency (Portable, Melbourne, Australia) was contracted to develop the digital health intervention. They used the human-centered Double Diamond approach to structure the design process [23]. Their team included digital producers, researchers, user experience designers, content strategists, and developers. During the primary period of development (July 2021 to February 2022), weekly web-based workshops were held with the various stakeholders. The aim of these workshops was three-fold: (1) to showcase developments and findings from interviews and consultations, (2) to discuss the content and key features that would be included in the intervention to ensure a process of iterative feedback and design, and (3) to ensure that the developed MVP was clinically applicable and had a minimal burden on clinical processes to facilitate successful implementation. A primary decision made during the iterative co-design workshops was that the website would be translated into Vietnamese, given that Vietnamese is the most widely spoken language at home, other than English, in the western suburbs of Melbourne [22,24]. The translation of the website into Vietnamese could then be used as a proof of concept to translate the website into other languages that are commonly spoken within the western suburbs of Melbourne.

### Developing an Evidence-Based Intervention

From early 2021, the literature was reviewed, and potential module topics and subtopics were collated by members of the research team with expertise in relevant fields (exercise physiology, physiotherapy, psychology, and dietetics). Key literature used to guide the content of the intervention included systematic reviews, meta-analyses, and clinical practice guidelines on osteoarthritis management [8,9,17,25]. The list of potential topics was inclusive of all disciplines found to be relevant to the primary outcome of improving the physical and mental health of patients with osteoarthritis. From August to November 2021, academics with expertise in a field relevant to osteoarthritis management were identified through Victoria University or professional networks and invited to provide feedback (written or verbal) on the development of the content. The academics had expertise in the following fields: exercise physiology, dietetics, psychology, physiotherapy, social work, osteopathy, pharmaceuticals, and public health. The collated information from published peer-reviewed literature was distributed among the academics over several iterative rounds to refine the content and recommended activities. The feedback from the collaborative process provided the opportunity to identify how the relevant fields build from one another to address the concerns of people living with osteoarthritis.

### Clinician Interviews

A total of 16 clinicians were recruited through Western Health and the networks of the research team. The clinicians were chosen based on their experience working with a range of patients in the identified LGAs as well as their experience working with people with osteoarthritis. Their profession, gender, and years of experience are presented in Table 1. A total of 11 structured interviews were conducted one to one or in small groups (2-3 people) in July and August 2021, each for a 1-hour duration. The aim of the interviews was to gain an understanding of the attitudes, beliefs, motivations, behaviors, and challenges of clinicians in relation to managing, treating, and interacting with people living with osteoarthritis. Questions related to the osteoarthritis treatment process focused on the characteristics of the patient population, treatment strategies, treatment efficacy in practice, and engagement of patients in the treatment process, among others.

**Table 1:** Demographics of the participants involved in the co-design process (N=42).

Demographics				Values
**Clinicians** **(n=16), n (%)**				
	**Gender**			
		Women	10 (63)	
		Men	6 (38)	
	**Profession**			
		Physiotherapist	6 (38)	
		Occupational therapist	3 (19)	
		Psychologist	2 (13)	
		Language services manager	1 (6)	
		General practice adviser	1 (6)	
		Rheumatologist	1 (6)	
		Orthopedic surgeon	1 (6)	
		Osteopath	1 (6)	
	**Years of clinical experience**			
		5-10	2 (13)	
		10-20	7 (44)	
		>20	7 (44)	
**End users involved in co-design interviews** **(n=11)**				
	**Gender, n (%)**			
		Women	7 (64)	
		Men	4 (36)	
	Age (years), mean (SD)		64.3 (7.2)	
	**Preferred language, n (%)**			
		English	5 (45)	
		Vietnamese	5 (45)	
		Greek	1 (9)	
	**Primary joint affected, n (%)**			
		Knee	6 (55)	
		Hip	3 (27)	
		Spine	1 (9)	
		Ankle	1 (9)	
**End users involved in feasibility testing (n=15)**				
	**Gender, n (%)**			
		Women	12 (80)	
		Men	3 (20)	
	Age (years), mean (SD)		61.5 (9.7)	
	**Country of birth, n (%)**			
		Australia	9 (60)	
		Vietnam	3 (20)	
		Chile	1 (7)	
		Philippines	1 (7)	
		New Zealand	1 (7)	
	**Preferred language, n (%)**			
		English	13 (87)	
		Vietnamese	2 (13)	
	**Education level, n (%)**			
		Did not complete secondary school	4 (27)	
		Completed secondary school	3 (20)	
		TAFE^a^ or trade certificate	3 (20)	
		Diploma or advanced diploma	3 (20)	
		Bachelor’s degree	2 (13)	
	**Primary joint affected, n (%)**			
		Knee	11 (73)	
		Hip	3 (20)	
		Both	1 (7)	

^a^TAFE: technical and further education.

### End User Interviews

Structured interviews were conducted with end users from the orthopedic waitlist at Western Health. The interviews were conducted to understand the needs, motivations, and barriers of end users regarding a digital health intervention. The inclusion criteria were as follows: diagnosed with osteoarthritis (by a general practitioner or the project physiotherapist), aged >18 years, and able to provide informed consent. The participant demographics are presented in Table 1. Members of the research team examined the records of patients to recruit end users who were representative of people with osteoarthritis (joint affected and age) as well as individuals from several of the most common cultural and linguistic groups in the western suburbs of Melbourne. In total, 2 rounds of interviews were completed with 11 end users. All interviews were conducted one to one and were 1 hour in duration. In 4 of the interviews, a family member was present to assist with language barriers and the communication of information. Of the 11 participants, 2 (18%) completed both rounds 1 and 2. Round 1 of interviews was completed in August 2021 with an early wireframe prototype of the intervention. End users answered questions about the high-level concepts and features in the intervention as well as the background of their condition and its management. Round 2 of interviews was completed in September and October 2021 and conducted with both English and Vietnamese speakers. A certified interpreter conducted the interviews in Vietnamese. In the second round of interviews, the end users were asked to engage with the mid-fidelity prototype and to provide feedback on the usability of the designs and their thoughts on content and features (Figure 1).

### Feature Prioritization

In October 2021, key stakeholders completed a prioritization workshop to select the features that would be included in the initial MVP product. The chosen features centered on creating and managing goals; accessing, managing, and editing content in the content management system; and accessing user analytics.

### Content Development

Intervention content was written and refined by the research team with the assistance of a content strategist to ensure that the content was delivered in simple English that was appropriate for the end users. This pragmatically involved limiting the amount of text as well as removing metaphors and ambiguity in the text where possible. Content templates were used to ensure consistency between modules. Anatomical illustrations, flowcharts, and figures were created, and images were used to ensure that the content was engaging and to assist in its comprehension. Audiovisual content was developed, filmed, and edited by the research team and media and film professionals. The video content included introductory videos, exercise demonstrations, and educational videos, which featured clinicians and academics. The audio content included mindfulness and relaxation exercises developed by the research team.

### Functionality Development

Interactive elements were incorporated within the intervention to increase user engagement. These included the options to set individualized goals and choose priority areas. Participants were encouraged to complete check-ins, which prompted them to modify or complete their goals and rate their progress in their priority areas. Progressive exercise programs were also developed by exercise professionals on the project team and included video- and text-based guidance for end users to follow each week.

### Translation

A bilingual member of the research team who read and spoke both English and Vietnamese fluently translated the content into Vietnamese. The translations were independently checked and modified as required by external National Accreditation Authority for Translators and Interpreters–certified translators. National Accreditation Authority for Translators and Interpreters certified members of the Western Health language services team and then reviewed the final translated documents.

### User Interface Design

The creation of a visual identity for the website was part of the design process. A combination of logo, color, typography, and iconography was developed during the design process and then applied to the interface. To build user confidence in the intervention, it was branded according to the official color palette of Western Health. To further increase user confidence, the logo was cobranded to represent both Victoria University and Western Health. End users provided input on the user interface design throughout the co-design process.

### Development and User Acceptance Testing

The development of the website was completed in December 2021, and a prototype was created for user acceptance testing. Immediate feedback from the research team was incorporated, and from January to February 2022, the MVP was released to the stakeholders for further user acceptance testing. All identified defects were rectified before the intervention was made available for testing with the end users.

### The Coventry, Aberdeen, and London–Refined Taxonomy of Behavior Change Techniques

An important aspect of our intervention is that the content contained evidence-based information that was theory driven from a behavior change perspective, incorporating evidence-based behavior change strategies (eg, goal setting and monitoring) [26], and was designed to maximize a patient-centered positive user experience. The content was designed such that users can set personalized goals and have autonomy over the way they engaged with it. We codeveloped the digital health intervention following the principles of the Coventry, Aberdeen, and London–Refined (CALO-RE) taxonomy of behavior change techniques [26].

### Feasibility Testing

From May to August 2022, the feasibility of the intervention was tested with 12 end users with osteoarthritis from the Western Health orthopedic outpatient waitlist. All patients, except for 1 (8%), were different from those who previously engaged in the co-design process. The patients were given 2 weeks to test the intervention and asked to explore the website, test the features, and try the activities. All participants received 2 semistructured check-in phone calls from the same member of the research team, during which they were provided technical assistance and their initial opinions of the intervention (design, functionality, and usability) were gauged. The first call occurred 1 to 2 days after receiving the on-boarding details, and the second call occurred 1 week into the testing period. At the conclusion of the 2-week testing period, the participants were asked to complete the System Usability Scale (SUS) to assess the usability of the intervention. The SUS includes 10 questions, with each answered on a 5-point Likert scale. The individual item scores were summed to provide a single overall score between 0 and 100, with 0 representing the worst possible score and 100 representing the best possible usability score [27].

## Results

### Outcomes of Developing an Evidence-Based Intervention

**Figure 2:**
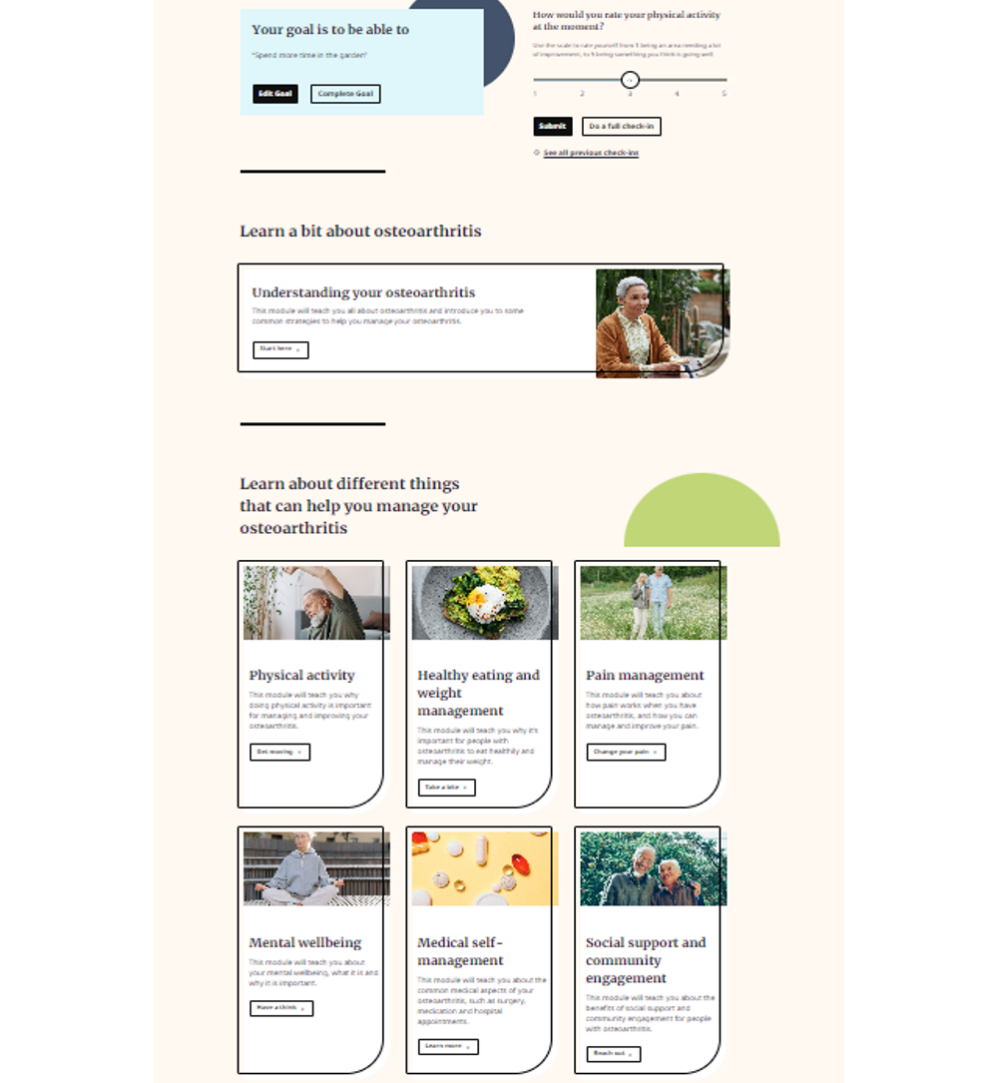
Screenshot of the home page.

A total of 7 key topics for the intervention modules were identified through a literature review. Table 2 summarizes the key concepts and features of these modules. Figure 2 is a screenshot of the home page showing the modules.

**Table 2:** Summary of the digital health intervention modules and curriculum developed during the co-design process.

Module title	Outline	Key concepts	Activities and resources
Understanding your osteoarthritis	General advice and information about osteoarthritis as a condition	Osteoarthritis is a condition involving the whole joint affected Recommended osteoarthritis management strategies	Goal-setting process with cues and functionality to facilitate check-ins on progress and reflection
Physical activity	Guidance about remaining physically active and reducing inactivity	The importance of remaining physically active for osteoarthritis Pacing and gradual progression of physical activity levels	Graded exercise program for hip and knee osteoarthritis that contained demonstration videos and was individualized to the current level of physical activity
Healthy eating and weight management	Dietary guidance and encouragement to lose weight if overweight	Importance of weight management for osteoarthritis Strategies to improve eating habits	Recipe bank with a variety of healthy meals from various cultural backgrounds
Pain management	Pain neuroscience education and active management strategies	Pain is not always a sign of harm Long-term strategies to help reduce pain	Guided relaxation audio (deep breathing and active PMR^a^)
Mental wellbeing	Advice on maintaining mental well-being while living with a chronic condition	The interaction between mental and physical health Strategies that can be used to improve mental well-being	Guided mindfulness audio recordings CBT^b^ or thought management worksheets
Medical self-management	Guidance for making the best health care decisions for people with osteoarthritis	Surgery recommended only for a small proportion of osteoarthritis cases Recommended and nonrecommended pharmaceutical treatments for osteoarthritis management	N/A^c^
Social support and community engagement	Sharing the benefits of social support and community engagement for people with osteoarthritis	Importance of support from friends, family, and community for managing osteoarthritis Identification of places and organizations where people can find community support	N/A

^a^PMR: progressive muscle relaxation.

^b^CBT: cognitive behavior therapy.

^c^N/A: not applicable.

### Outcomes of Clinician Interviews

The insights from the interviews with the clinicians are reported in Textbox 1. Key decisions made based on these findings included the use of multiple content delivery formats (written text, videos, audio, and images) to support those with low literacy levels and provide different learning options. In addition, the involvement of clinical staff from Western Health and academics from Victoria University in videos was a strategy used to enhance the trustworthiness of the content and support the perceived hierarchical structure of practitioners. Finally, individualized goal setting and check-in functions were included to allow end users to visualize their priorities and track their progress.

**Key insights gained from clinician interviews.**

**What are the characteristics of patients in the western suburbs of Melbourne, Australia?**

- Patients come from a range of culturally and linguistically diverse backgrounds.
- Patients possess variable socioeconomic, health, digital, and written literacy levels.
- There is a high likelihood of patients having comorbidities.
- “If I describe the cohort in western area patient group, it’s a very diverse kind of group” (C07).

**Misunderstandings of health care and services**

- A common belief is that surgery is the only treatment for osteoarthritis.
- Patients’ belief of a hierarchy among medical experts (surgeons at the top, followed by general practitioners [GPs] and then allied health practitioners) negatively impacts their engagement with allied health practitioners.
- “I think in terms of engagement when people are referred to allied health or services from allied it’s often a second priority for them. They’ve seen a GP or surgeon and they’ve been referred on and it’s not the number one outcome they were expecting” (C07).

**Patient-driven approach and engagement**

- Communication of treatment benefits needs to resonate with the patient.
- A key barrier to accepting conservative treatment is that people assume that when they experience pain it is because they are causing damage.
- “I say to them on the first day if you do this the surgery outcome will be better, so they know from the very beginning they’re not wasting their time” (C15).

**Tailored goal setting and assessment**

- Making goals specific to a person’s life increases the chance of adherence.
- Assessing diverse outcomes is important (function and symptoms).
- Frequent check-ins are important in early stages for effective behavior change.
- “Our key role is working hard to understand what a person’s normal life is like, how they compensate, what stops them doing what they want, their environment and important support roles in their life. We’ll have young guys who say they can’t get up, but when they need a smoke, they’ll be hopping down stairs. We need to understand what’s important to them because it drives therapies, helps them understand and gets better buy in” (C11).

**Suggestions for tailoring content and increasing engagement with resources**

- Starting with approaches and methods patients are familiar with is recommended.
- Resources need to be reputable and available at times when patients need them.
- A range of mediums are used to communicate information based on what works best for each patient.
- “Give patients as many tools as you can and find out what works for them” (C16).

### Outcomes of End User Interviews

The insights gained from the interviews with the end users are presented in Textbox 2. A key finding from the end user interviews was that the participants wanted the intervention to be individualized to them and specific to their needs. This meant that the intervention needed to be developed for people with knee and hip osteoarthritis specifically and that different exercise programs needed to be developed for the knee and hip. Other key findings included the need for the intervention to come from a trustworthy source, the need to be able to access the intervention content in a variety of formats, and the importance of providing patients with information early in their treatment journey.

**Key insights gained from end user interviews.**

**Experiences of living with osteoarthritis**

- Round 1
  Progression of osteoarthritis leads to the loss of independence and reliance on others.
  Osteoarthritis leads to feelings of frustration, depression, and helplessness.
  “Pretty determined I just will do it then suffer the pain later but now I can’t do that so just giving into it...it can be really depressing and life changing and not only affects me but my husband and children and grandchildren...can’t even go on the bike with them”(P04).

**Self-management experiences**

- Round 1
  Various self-management techniques are used, and it is hard to stay consistent.
  Information is obtained from the internet, friends, family, or medical professionals.
  Internet was often overwhelming, unstructured, and unguided.
  “...just managing it, I’ve lost a little bit of weight, that helped a bit but with all these lockdowns it comes back on. I get bored and then you have a bit of a chomp on something” (P03).

**Perceived benefits of the digital health intervention**

- Round 1
  The intervention provides access to trustworthy information from a local health service.
  The intervention can lead to an improvement in symptoms and a reduction in pain.
  The website can assist the end users in a variety of ways.
  “It’s reassuring that it’s the western suburbs of Melbourne, so you know it’s relevant to our health services” (P01).

**Reflections on the content of the digital health intervention**

- Round 1
  The content should be specific to each patient.
  The content should give people insights into what is normal.
  “Yeah, if you’ve got to go through neck, back, hands, fingers everything else before the knee you’re not gonna do it...” (P04).
- Round 2
  Audiovisual content is the most beneficial, but it is also helpful to have written content.
  Having integrated topics may help draw connections for a more holistic understanding of self-management.
  “He doesn’t want to read about it he wants someone to show him, like a video, how to reduce the pain” (P05).

**Experiences of goals setting and tracking in the digital health intervention**

- Round 1
  Goal setting and tracking provide motivation and help understand progress.
  Goal setting helps people set simple and realistic goals.
  “Yeah, I suppose if you don’t set that goal and baseline, you don’t know how far you’ve come from that baseline” (P01).
- Round 2
  Example goals are useful for engaging people and help people understand the purpose of the intervention.
  Prompting self-assessments allows reflection and removes reliance on self-nomination.
  “It’s good that there are examples, but also that you give helpful examples to choose from” (P07).

**Considerations for engaging with the digital health intervention**

- Round 1
  Timely access to educational information about osteoarthritis and its management in the health care journey is important.
  Purpose of the website and content should be clear.
  “[The] thing that was interesting [on the website] is that it says it can make surgery unnecessary and reduce your pain and all that...anything to avoid going under the knife would be good...bit hard with arthritis as it’s bone on bone and in the joints” (P03).
- Round 2
  The earlier in the treatment journey patients have access to educational information about osteoarthritis and its management, the better.
  Some people with osteoarthritis may have barriers to accessing the intervention, and it is possible that carers may be the ones that access it.
  Language barriers may not just be a functional barrier but may impact someone’s desire to engage with the intervention.
  “I was looking through last night and thinking I wish I had known all this before” (P02).

### The CALO-RE Taxonomy of Behavior Change Techniques

Our digital health intervention was informed by the effective behavior change techniques in the CALO-RE taxonomy. We identified many of these techniques as feasible for the intervention and adopted them, as outlined in Textbox 3.

**Coventry, Aberdeen, and London–Refined taxonomy behavior change techniques used in the intervention.**

**Information provision (general)**

General health information is provided across 7 modules addressing various aspects of osteoarthritis management.

**Goal setting (behavior and outcome)**

Patients are encouraged to set individualized goals upon commencing the intervention.

**Review of behavioral goals or outcome goals**

Patients have the opportunity to review their progress, modify goals, and create new goals.

**Informing when and where to perform behavior**

Written and audio information about when and where to complete healthy behaviors, such as physical activity, mindfulness, and relaxation activities, is provided.

**Instruction on how to perform the behavior**

Written and audio information on how to complete healthy behaviors, such as exercises, recipes, mindfulness, and relaxation activities, is provided.

**Demonstrate behavior**

Videos and images are used to demonstrate how to complete exercises and recipes.

**Training to use prompts**

Education is provided on the use of reminders and diaries to facilitate adherence to health behaviors.

**Plan social support**

Information is provided on the benefits of social support, and various local support groups are listed in intervention.

**Fear arousal**

Patients are informed of the health risks that are commonly associated with osteoarthritis and what may occur if they do not implement behavioral changes.

**Stress management**

Information about stress and activities that help reduce stress is provided in the intervention.

**Stimulate the anticipation of future rewards**

Patients are informed of the physical and mental health rewards they would get from implementing the health behaviors.

### Feasibility Testing

The results of the feasibility testing are presented in Table 3. Of the 15 participants, 2 (13%) dropped out of the testing: 1 before the first check-in phone call, and 1 before the second check-in phone call because of unplanned commitments (work and personal reasons) and a loss of communication. A further participant completed the second check-in phone call but not the first check-in phone call, so in total 13 individuals participated in each check-in phone call. The mean SUS score of 82.7 (SD 16) places the usability of the intervention in the excellent category, meaning that the participants found the intervention effective and easy to use. The responses captured during the check-in phone calls suggest that the participants accessed the intervention on a range of devices and that the website was trustworthy, appealing, and required minimal technical support.

**Table 3:** Results of the feasibility testing (n=13)^a^.

				Values
SUS^b^ score, mean (SD)				82.7 (16)
**Check-in call 1 (responded yes), n (%)**				
	Does the website seem interesting and appealing?^c^			11 (92)
	Was the log-in and goal-setting process easy?^c^			12 (100)
	Does the website appear trustworthy?			13 (100)
	Was troubleshooting or support needed?			1 (8)
**Check-in call 2 (responded yes), n (%)**				
	**Devices used**			
		Desktop	4 (31)	
		Laptop	5 (38)	
		Smartphone	4 (31)	
		Tablet	2 (15)	
		Multiple	2 (15)	
	Do the features of the website seem appealing and interesting? n (%)^d^			10 (91)
	Was troubleshooting or support needed? n (%)			12 (92)

^a^Of the participants, 2 (13%) dropped out and a further participant completed the second check-in phone call but not the first check-in phone call, so in total 13 individuals participated in each check-in phone call.

^b^SUS: System Usability Scale.

^c^N=12 as only 12 participants responded.

^d^N=11.

## Discussion

### Principal Findings

A digital health intervention was successfully co-designed and an MVP was developed to provide evidence-based advice to patients with osteoarthritis. The feasibility of the intervention was tested with end users, who identified that the intervention was appealing and easy to use. This patient- and clinician-informed solution has been designed such that it can be easily implemented in a clinical setting and has been developed within a framework of the CALO-RE taxonomy of behavior change techniques [28]. The demographics of the population living in the western suburbs of Melbourne (languages spoken, cultural diversity, education levels, income, and health literacy) were used to inform our patient-centered approach to developing the intervention [6,22]. A key potential benefit of this intervention is that it will provide access to relevant and evidence-based health advice for people who often have limited access to health services. The intervention enables patients to set individualized goals; access information and education in video, visual, and written formats; and complete activities designed to help them manage their osteoarthritis symptoms and improve their quality of life.

### Comparison With Previous Work

The major difference between this intervention and the previously developed digital interventions for people with osteoarthritis is that this intervention was co-designed with the end users. The inclusion of patients from CALD backgrounds was unique. This study considered not only the language and cultural perspectives but also the social status and health, digital, and written literacy of the population. This was important to understand, as many previous studies excluded participants who did not speak the native language of the country in which the interventions were developed [29-33]. Our approach enabled nuanced and multiperspective practical recommendations and the development of an intervention suitable to the population group serviced by the local health service. The type of content provided by previous digital interventions for people with osteoarthritis is variable but is usually focused on the core topics recommended for the management of osteoarthritis [16]. Physical activity education and exercise programs comprise the primary content provided by most interventions. Some provide physical activity content alone [29,34,35], some provide physical activity content along with osteoarthritis-specific education [30,36,37], and others further include nutrition and weight management advice [31,32,38,39]. The content provided in this intervention included all the core recommended osteoarthritis treatment strategies, namely physical activity; weight management; osteoarthritis-specific education; information and resources about mental health and well-being, the medical management of osteoarthritis, and the importance of social support; and pain neuroscience education.

### Intervention Implementation

The website will be updated based on the feedback provided in the current feasibility study. Following the completion of a randomized controlled trial to examine the intervention’s effectiveness in improving the health and well-being of patients and cost-effectiveness, it is hoped that the intervention will be implemented as part of usual care in the clinical setting. It is envisaged that if the intervention is implemented in clinical care, patients who are added to the orthopedic waiting list will be provided with a link to the intervention as part of usual care.

### Limitations

Owing to financial and pragmatic constraints, the digital health intervention was developed only in English and Vietnamese. Although many other linguistic and cultural groups reside in the western suburbs of Melbourne, the inclusion of 1 CALD community served as a proof of concept for the capability to effectively translate the intervention. In our future work, we aim to expand the number of languages available on the website to cater to more of the target population. Despite this, the use of videos, audio, and images may allow people with low English-language proficiency and literacy levels to engage with the content in a meaningful manner. A potential bias of the study is that several authors were also involved as participants in the structured interviews.

### Recommendations for Future Research

Several key recommendations from this study will assist in the development of future digital health interventions. First, when engaging with and recruiting participants from CALD backgrounds, it is important to ensure that all communication and documentation are available in their preferred language. Second, patients are more likely to trust information if it is provided by a credible and local source. Finally, developing an intervention that is individualized as much as feasible will provide patients with confidence that the intervention is specific to them and their condition.

### Conclusions

The digital health intervention has been designed to be a potentially viable and freely accessible means of supporting individuals with osteoarthritis while they are on the waiting list for an orthopedic specialist consultation. The intervention uses a holistic, evidence-based approach to improve patient-centered care. It was developed specifically for patients in the western suburbs of Melbourne, Australia. However, the principles and approach adopted to develop the intervention can be applied across geographical and cultural settings to enhance access to evidence-based strategies to manage osteoarthritis while waiting for specialized care. The effectiveness of the intervention in improving the physical and mental health of patients will subsequently be determined through a high-quality randomized controlled trial.

## Abbreviations

CALD: culturally and linguistically diverse
CALO-RE: Coventry, Aberdeen, and London–Refined
LGA: local government area
MVP: minimum viable product
SUS: System Usability Scale

## Data Availability

The deidentified data sets generated during this study are available from the corresponding author upon reasonable request.
